# Ubiquitination of SARS-CoV-2 ORF7a Prevents Cell Death Induced by Recruiting BclXL To Activate ER Stress

**DOI:** 10.1128/spectrum.01509-22

**Published:** 2022-11-03

**Authors:** Zhixin Liu, Yanan Fu, Yanping Huang, Feng Zeng, Jingjing Rao, Xiao Xiao, Xiaoguang Sun, Hao Jin, Jian Li, Jing Yang, Weixing Du, Long Liu

**Affiliations:** a Department of Infectious Diseases, Renmin Hospital, School of Basic Medical Sciences, Hubei University of Medicine, Shiyan, China; b Institute of Virology, Hubei University of Medicine, Shiyan, China; c Hubei Key Laboratory of Embryonic Stem Cell Research, Hubei University of Medicine, Shiyan, China; Indian Institute of Science Bangalore

**Keywords:** ORF7a, SARS-CoV-2, ER stress, apoptosis, ubiquitination

## Abstract

Severe acute respiratory syndrome coronavirus 2 (SARS-CoV-2) is the causative agent of coronavirus disease 2019 (COVID-19), which has emerged in the last 2 years. The accessory protein ORF7a has been proposed as an immunomodulating factor that can cause dramatic inflammatory responses, but it is unknown how ORF7a interacts with host cells. We show that ORF7a induces cell apoptosis by recruiting the prosurvival factor BclXL to the endoplasmic reticulum (ER) via the exposed C-terminal residues Lys117 and Lys119. Simultaneously, ORF7a activates ER stress via the PERK-elF2α-CHOP pathway and inhibits the expression of endogenous BclXL, resulting in enhanced cell apoptosis. Ubiquitination of ORF7a interrupts the interaction with BclXL in the ER and weakens the activation of ER stress, which to some extent rescues the cells. Our work demonstrates that SARS-CoV-2 ORF7a hires antiapoptosis protein and aggregates on the ER, resulting in ER stress and apoptosis initiation. On the other hand, ORF7a utilizes the ubiquitin system to impede and escape host elimination, providing a promising potential target for developing strategies for minimizing the COVID-19 pandemic.

**IMPORTANCE** Viruses struggle to reproduce after infecting cells, and the host eliminates infected cells through apoptosis to prevent virus spread. Cells adopt a special ubiquitination code to protect against viral infection, while ORF7a manipulates and exploits the ubiquitin system to eliminate host cells' effect on apoptosis and redirect cellular pathways in favor of virus survival. Our results revealed that SARS-CoV-2-encoded accessory protein ORF7a recruits prosurvival factor BclXL to the ER and activates the cellular ER stress response resulting in the initiation of programmed death to remove virus-infected cells. Ubiquitination of ORF7a blocked the recruitment of BclXL and suppressed the ER stress response, which helps to counteract cell apoptosis and rescue cell fate. These findings help us understand the mechanism of SARS-CoV-2 invasion and contribute to a theoretical foundation for the clinical prevention of COVID-19.

## INTRODUCTION

SARS-CoV-2 has brought about an ongoing pandemic of COVID-19. However, the complete mechanism of SARS-CoV-2 infection and pathogenicity is far from clear. Apoptosis has been observed in different infected tissues, such as the lung, liver, testis, thyroid, and spleen, obtained during autopsy studies on SARS-CoV or SARS-CoV-2 casualties (1–4). It has been reported that SARS-CoV ORF7a can induce caspase-dependent apoptosis in cells derived from the lung, liver, and kidney (5, 6). Studies have also indicated that ORF7a-induced apoptosis depends on its interaction with the antiapoptosis factor BclXL (7). The SARS-CoV-2 ORF7a protein shares 95.9% sequence similarity and 85.2% identity with this protein from SARS-CoV (8), leading to wonder whether SARS-CoV-2 ORF7a also has proapoptotic activity.

Apoptosis regulates the host's inflammatory response. It is also very important for the host to defend against and control viral infection (9). Lymphopenia is one of the most common clinical symptoms of COVID-19 and is caused by T lymphocyte apoptosis (10). Severe COVID-19 is characterized by extensive T cell dysfunction and apoptosis (11), and this antagonism of cell homeostasis and apoptosis is also present in other infected cells.

The B cell lymphoma-2 (Bcl-2) family plays an important role in apoptosis. Some members of the Bcl-2 family are prosurvival, such as BclXL, whose overexpression inhibits apoptosis induced by viral infection (12, 13). For example, some viruses encode proteins, such as herpesvirus-encoded Nr-13, that are functional homologs of cellular Bcl-2 proteins and inhibit apoptosis and persist in host cells for the benefit of viral replication (14, 15). Bcl-2 inhibits SARS-CoV infection-induced caspase-dependent apoptosis without affecting viral replication kinetics (16). One study reported that SARS-CoV E protein induces apoptosis, inhibited by BclXL overexpression (17).

SARS-CoV-2 accessory proteins play critical roles in virus-host interactions and the modulation of host immune responses, thereby contributing to coronaviral pathogenicity via different strategies (18). However, the functions of the SARS-CoV-2-encoded accessory protein ORF7a are still largely unknown. Here, we found that ORF7a localizes on the endoplasmic reticulum (ER), and it is important to study why ORF7a rushes to the ER. Accumulating evidence has demonstrated that SARS-CoV's spike and ORF6 proteins could induce the ER stress response (19, 20). Determining whether SARS-CoV-2 ORF7a causes an ER stress response and the impact of this response on cell fate is an urgent need.

This study demonstrated that SARS-CoV-2 ORF7a recruits BclXL to the ER through the C-terminal positively charged amino acids Lys117 and Lys119, which weakens BclXL’s ability to inhibit cell apoptosis. Simultaneously, ORF7a activates the cellular ER stress response and enhances cell apoptosis. On the other hand, ubiquitination of ORF7a blocked the interaction with BclXL and suppressed ORF7a localization to the ER, resulting in weakened activation of ER stress and rescued cells. These findings help us understand the mechanism of SARS-CoV-2 invasion and contribute to a theoretical foundation for the clinical prevention of COVID-19.

## RESULTS

### SARS-CoV-2 ORF7a induces cell apoptosis.

**FIG 1:**
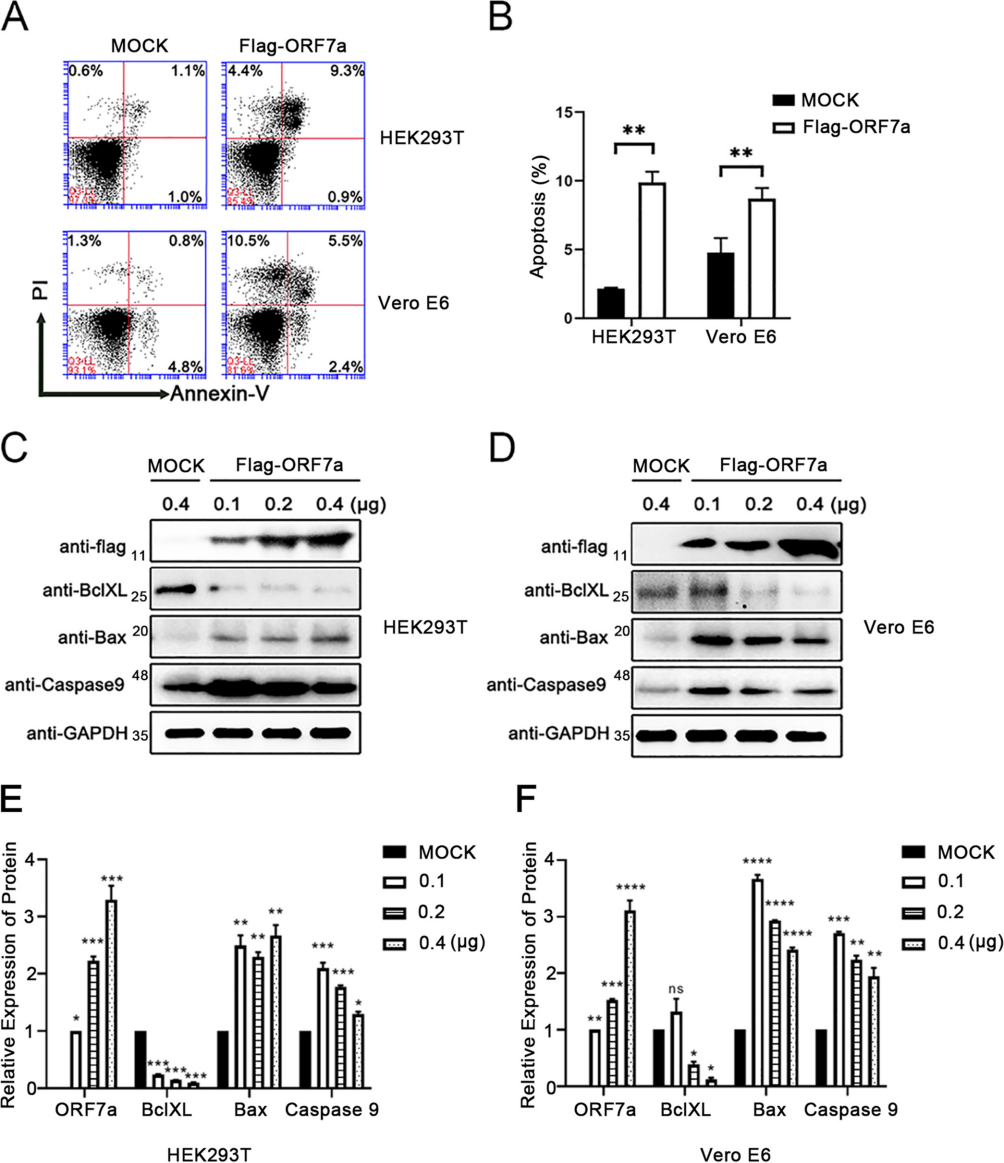
ORF7a induces cell apoptosis in HEK 293T and Vero E6 cells. (A) Apoptosis in HEK293T and Vero E6 cells was measured using flow cytometry at 24 h after transfection with pCAG-Flag (MOCK) or pCAG-Flag-ORF7a, respectively. (B) The percentage of cell apoptosis was quantified from three independent measurements. (C and D) ORF7a-Flag, endogenous BclXL, Bax, Caspase9, and GAPDH in HEK293T cells and Vero E6 cells were detected by Western blotting at 24 h after transfection with pCAG-Flag (MOCK) and pCAG-Flag-ORF7a. (E and F) ORF7a-Flag, BclXL, Bax, and Caspase9 Western blotting results were calculated for three independent experiments using ImageJ. **, *P < *0.05; **, *P* < 0.01; and ***, *P* < 0.001, compared with the negative-control group (one-way ANOVA and Tukey's *post hoc* test).

Accessory proteins of SARS-CoV-2 function in modulating host immune responses and contribute to pathogenesis. Recently, ORF7b has been verified by our lab to induce cell apoptosis (21). Here, we found that the adjacent ORF7a also has the same function in inducing apoptosis in HEK 293T and Vero E6 cells (Fig. 1A and B). Western blotting showed that the antiapoptosis protein BclXL decreased significantly with increasing ORF7a expression (Fig. 1C–F). The expression of the apoptotic protein BAX and protease Caspase 9 robustly increased when there was a low concentration of ORF7a (Fig. 1C and D). In addition, Bax and Caspase 9 expression decreased with the increasing ORF7a concentration in the two cell lines (Fig. 1C–F). Considering the antagonistic effect of BclXL on cell apoptosis, these results indicate that ORF7a induces a decrease in BclXL that results in cell apoptosis.

### ORF7a interacts with BclXL through the C-terminal residues Lys117 and Lys 119, and its proapoptosis activity is dependent on this interaction.

**FIG 2:**
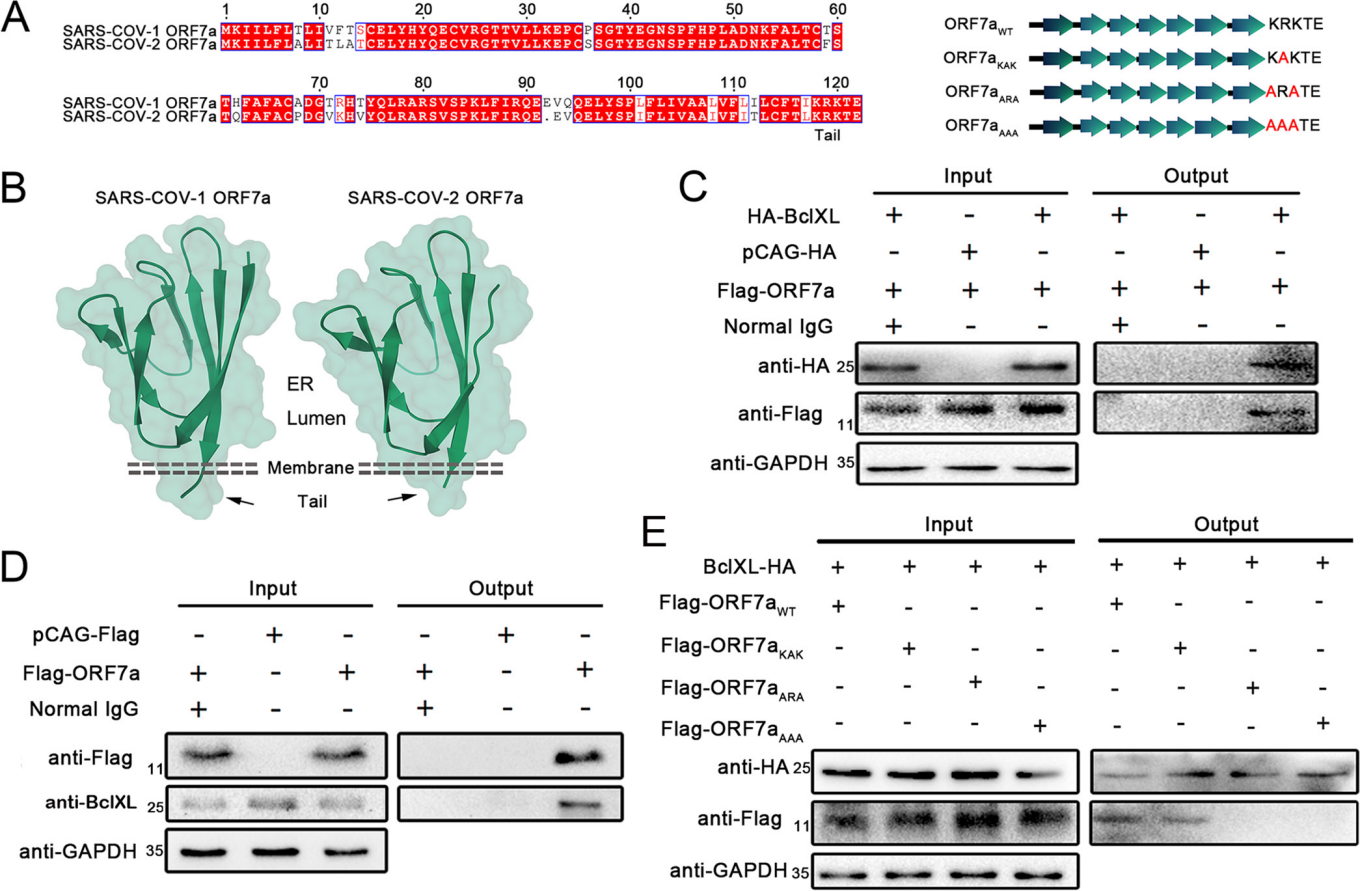
ORF7a interacts with BclXL through the Lys117 and Lys119 residues. (A) SARS-CoV-1 and SARS-COV-2 ORF7a sequences were compared using MEGA-X sequence alignment. ORF7a C-terminal mutants, ORF7a_ARA_, ORF7a_KAK_, and ORF7a_AAA_, were generated and compared. (B) Three-dimensional structures of SARS-CoV-1 and SARS-CoV-2 ORF7a were visualized and compared using PyMOL 1.0 software. (C) HEK293 cells were cotransfected with pCAG-HA-BclXL and pCAG-Flag-ORF7a for 24 h. The cells were lysed, and the lysates were immunoprecipitated with anti-HA antibodies. Immunoprecipitates and WCLs were analyzed via WB with the indicated antibodies. (D) HEK293 cells were transfected with pCAG-Flag-ORF7a for 24 h, and the endogenous BclXL interaction with Flag-ORF7a was confirmed by IP using an anti-Flag antibody. (E) HEK293 cells were cotransfected with pCAG-HA-BclXL, pCAG-Flag-ORF7a, or mutant (pCAG-Flag-ORF7a_KAK_, pCAG-Flag-ORF7a_ARA_, or pCAG-Flag-ORF7a_AAA)_ for 24 h. The cells were lysed, and the lysates were immunoprecipitated with anti-HA antibodies. Immunoprecipitates and WCLs were analyzed via WB with the indicated antibodies.

**FIG 3:**
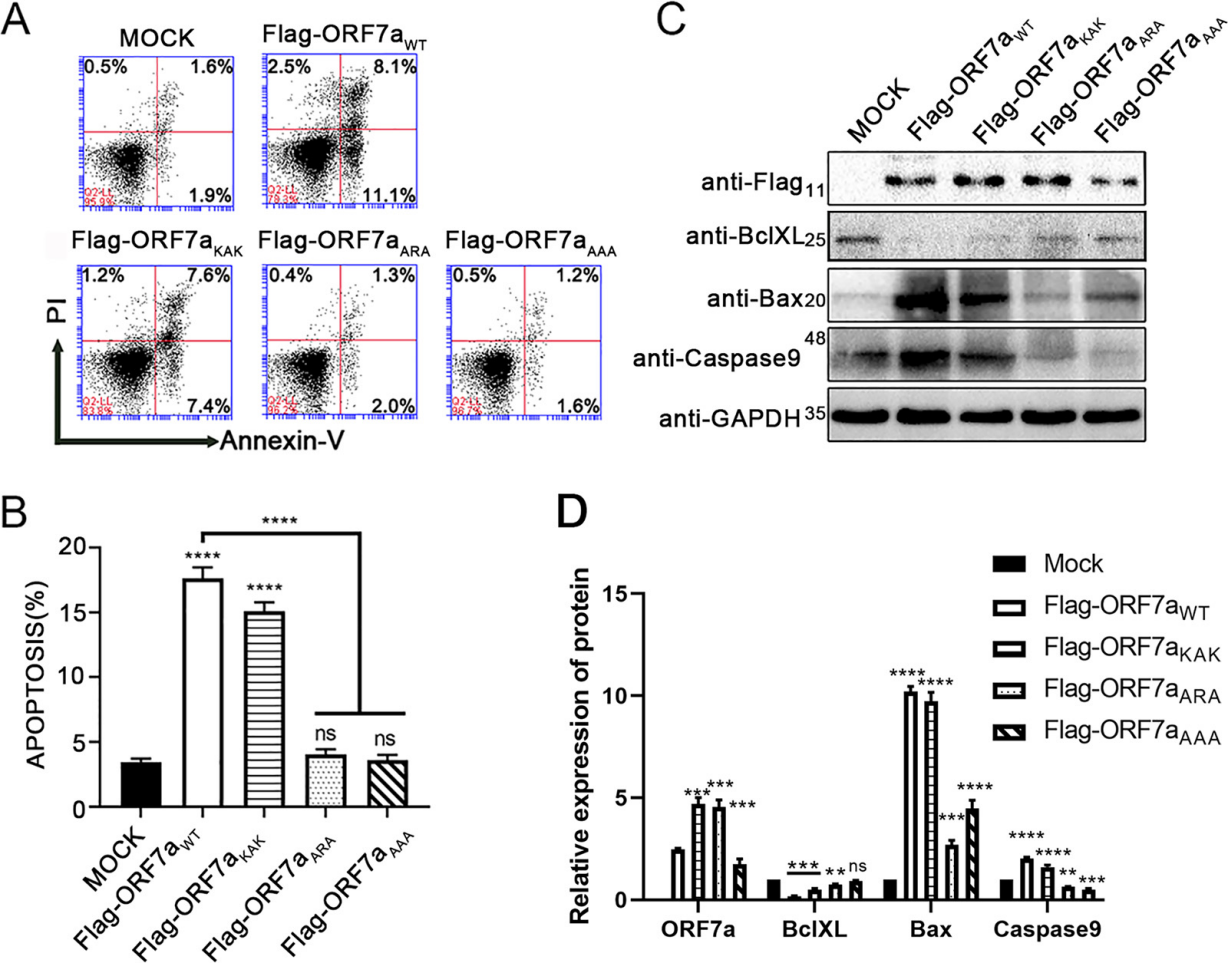
ORF7a mutants lost the ability to induce apoptosis. (A) Apoptosis was measured using flow cytometry at 24 h after transfection with pCAG-Flag (MOCK), pCAG-Flag-ORF7a, or mutant (pCAG-Flag-ORF7a_KAK_, pCAG-Flag-ORF7a_ARA_, or pCAG-Flag-ORF7a_AAA_). (B) The percentage of cell apoptosis was quantified from three independent measurements. (C) Protein expression of Flag-ORF7a, endogenous BclXL, Bax, Caspase9, and GAPDH in Vero E6 cells was detected by Western blotting at 24 h after transfection with pCAG-Flag (MOCK), pCAG-Flag-ORF7a, or mutant (pCAG-Flag-ORF7a_KAK_, pCAG-Flag-ORF7a_ARA_, or pCAG-Flag-ORF7a_AAA_). (D) Flag-ORF7a, BclXL, Bax, and Caspase9 Western blotting results were calculated for three independent experiments using ImageJ. *, *P* < 0.05; **, *P* < 0.01; and ***, *P* < 0.001, compared with the negative-control group (one-way ANOVA and Tukey's *post hoc* test). ns., no significant difference.

SARS-CoV-2-encoded ORF7a has 85.2% sequence identity and a structure similar to SARS-CoV ORF7a (Fig. 2A and B), which indicates a short cytoplasmic tail within the ER and Golgi network (22). A previous study reported that SARS-CoV-encoded ORF7a interacts with BclXL and interferes with its prosurvival function (7). Herein, through the immunoprecipitation assays, we found that SARS-CoV-2 ORF7a also possesses the ability to interact with BclXL, including overexpressed and endogenous BclXL (Fig. 2C and D). To identify the mechanism of specific binding between ORF7a and BclXL, the domain and extracellular region of ORF7a were analyzed, and we constructed three ORF7a mutants named ORF7a_KAK_, ORF7a_ARA_, and ORF7a_AAA_ (Fig. 2A). Coimmunoprecipitation assays showed that ORF7a_ARA_ and ORF7a_AAA_ interrupted the interaction with BclXL (Fig. 2E), indicating lysine residues at the C-terminal 117 and 119 sites exert critical roles in ORF7a-BclXL binding. To evaluate the function of the ORF7a-BclXL interaction in the process of ORF7a-induced cell apoptosis, the apoptosis rate was detected by flow cytometry. HEK 293T cells expressing mutated ORF7a showed a decreased apoptotic rate relative to the wild-type ORF7a group (Fig. 3A and B). Endogenous BclXL was decreased in the cells expressing wild-type or mutated ORF7a compared to the mock group (Fig. 3C and D). Bax and Caspase 9 increased significantly in the wild-type group but returned to lower levels in the ORF7a_ARA_ and ORF7a_AAA_ groups (Fig. 3C and D). These results confirm that the proapoptotic ability of ORF7a depends on the interaction with BclXL.

### ORF7a interacts with BclXL to change its subcellular localization.

**FIG 4:**
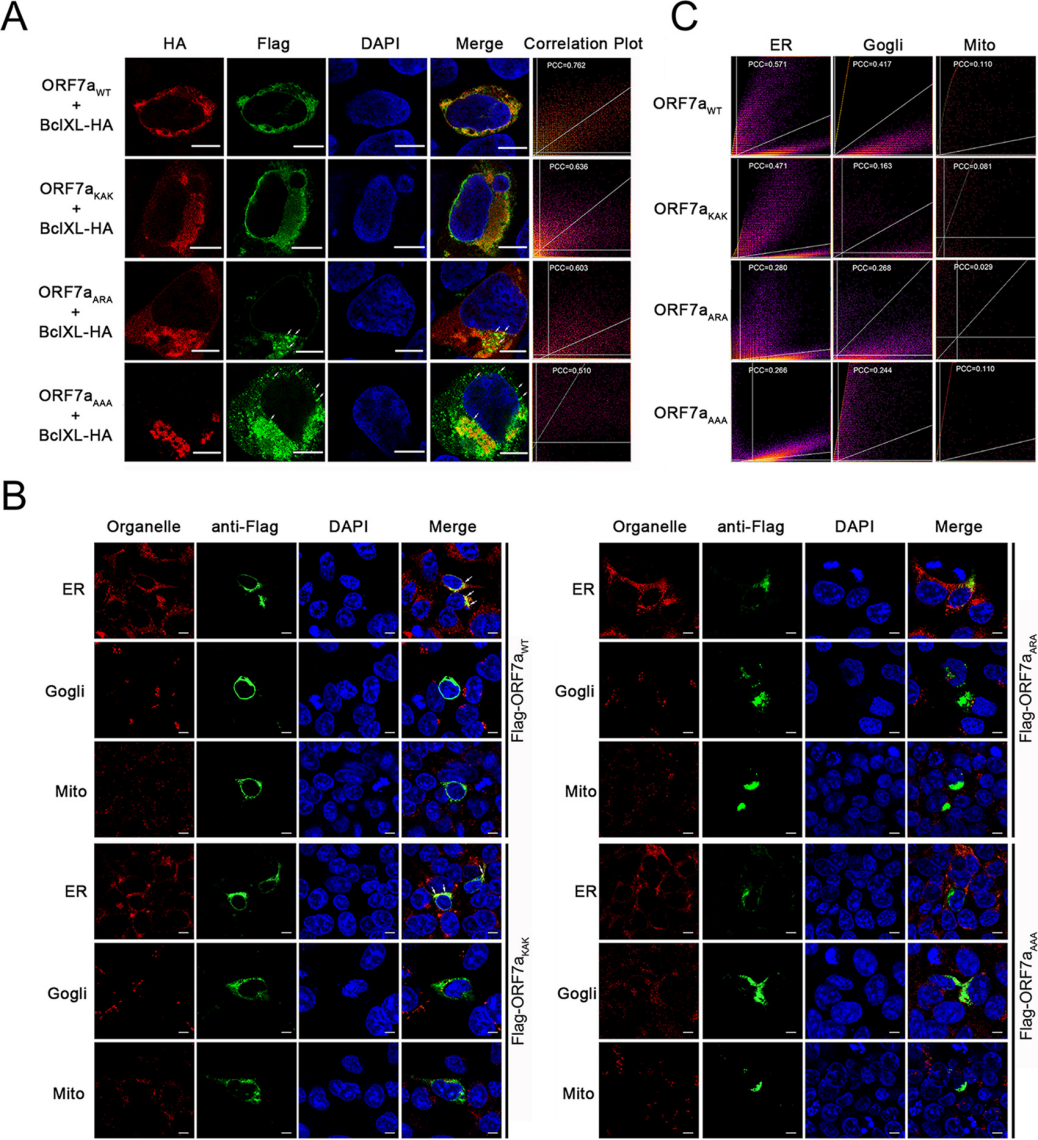
ORF7a interacts with BclXL to change its subcellular localization. (A) HEK293T cells were cotransfected with pCAG-Flag-ORF7a (or mutant) and pCAG-HA-tagged BclXL plasmids for 24 h. The cellular localization of ORF7a (or mutant) and BclXL was observed via confocal microscopy. Scale bar, 10 μm. (B) HEK293T cells were cotransfected with pCAG-Flag-tagged ORF7a (or mutant) plasmid for 24 h. Cellular localization of ORF7a (or mutant), the endoplasmic reticulum (ER), mitochondria, and the Golgi body were visualized via confocal microscopy. Scale bar, 10 μm. (C) Pearson's correlation coefficient (PCC) between the indicated proteins was calculated to determine whether the two proteins were colocalized.

To determine the proapoptotic mechanism of ORF7a after interacting with BclXL, we first observed the subcellular localization of ORF7a accompanied by BclXL. Immunofluorescence showed that wild-type ORF7a and ORF7a_KAK_ colocalized with BclXL and were scattered around the nucleus (Fig. 4A), but mutant ORF7a_ARA_ and ORF7a_AAA_ clustered in puncta and dotted in the corners of cytoplasm or outside the nucleus and did not colocalize well with BclXL. The Pearson's correlation coefficient (PCC) was between 0.5 and 1.0, indicating that the two fluorescence signals were colocalized (23). To clarify the subcellular localization of ORF7a, we stained the three organelles. As shown in Fig. 4B, wild-type ORF7a localized to the endoplasmic reticulum membrane rather than the Golgi or mitochondria, whereas ORF7a_KAK_ lost part of its localization to the endoplasmic reticulum (Fig. 4B and C). Although the mutants ORF7a_ARA_ and ORF7a_AAA_ displayed aggregation as plaques, they could not be observed to be fully colocalized with any of the three organelles (Fig. 4B and C). These results indicate that ORF7a interacts with BclXL through Lys117 and Lys119 and that the interaction recruits BclXL to the endoplasmic reticulum.

### ORF7a actives ER stress via the PERK-elF2α-CHOP pathway.

**FIG 5:**
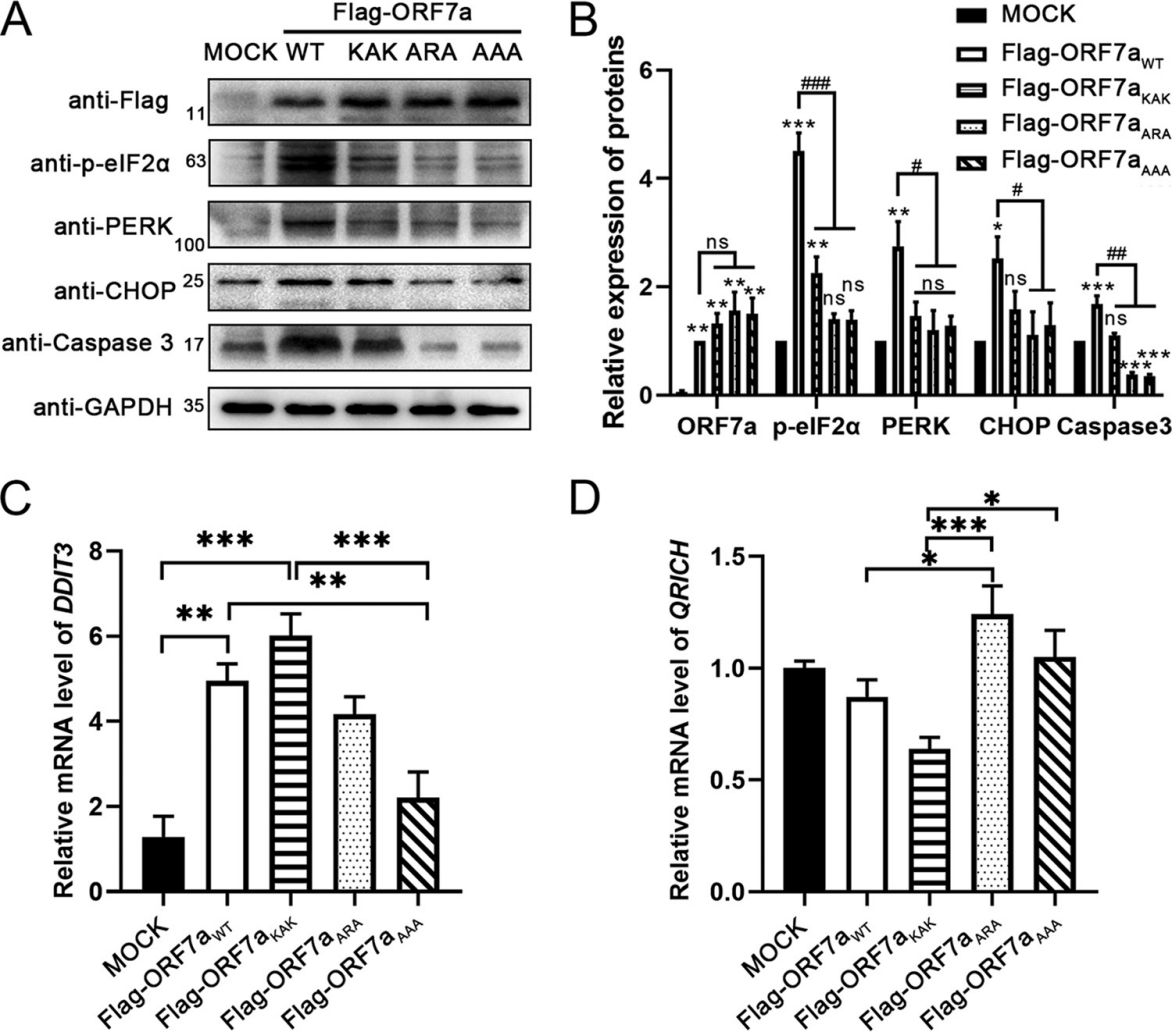
ORF7a actives ER stress via the PERK-elF2α-CHOP pathway. (A) Protein expression of ORF7a-Flag, p-eIF2a, PERK, CHOP, cleaved Caspase 3, and GAPDH in Vero E6 cells was detected by Western blotting at 24 h after transfection with pCAG-Flag, pCAG-Flag-ORF7a, or mutant (pCAG-Flag-ORF7a_KAK_, pCAG-Flag-ORF7a_ARA_, or pCAG-Flag-ORF7a_AAA_). (B) Calculation of ORF7a-Flag, p-eIF2a, PERK, CHOP, and cleaved Caspase 3 Western blotting results were performed for three independent experiments using ImageJ. (C and D) mRNA expression of *DDIT3* and *QRICH* in Vero E6 cells was determined by qRT–PCR at 24 h after transfection with pCAG-Flag, pCAG-Flag-ORF7a, or its mutants. *, *P* < 0.05; **, *P* < 0.01; and ***, *P* < 0.001, compared with the mock group. *#*, *P* < 0.05; #*#*, *P* < 0.01; and ##*#*, *P* < 0.001, compared with the WT group (one-way ANOVA and Tukey's *post hoc* test). ns., indicates no significant difference.

To further explore the function of ORF7a aggregation on the endoplasmic reticulum, we detected the expression of the cellular ER stress transducer protein kinase RNA-like ER kinase (PERK) and its downstream effectors elF2α and C/EBP homologous protein (CHOP). Notably, the expression of wild-type ORF7a located on the ER upregulated the ER stress transducer PERK. At the same time, ORF7a mutants weakened the activation with the subcellular location change (Fig. 5A and 4B). The effectors phosphorylated elF2α and CHOP were also upregulated in the wild-type ORF7a group. The mutations impaired the upregulation (Fig. 5A and B). Elevated cleaved PARP-1 in the wild-type group and weakened cleavage of PARP-1 in mutant groups also indicate the ER stress response (see Fig. S1A and C in the supplemental material). The mRNA level of *DDIT3*, which encodes CHOP, was also upregulated, like the protein level (Fig. 5C). *QRICH1*, a key effector of the PERK-eIF2a axis of the ER stress-triggered unfolded protein response (24), dictates cell fate in response to pathological ER stress. The mRNA level of *QRICH1* was not affected by treatment with wild-type ORF7a or its mutants relative to the mock group (Fig. 5D), although the mRNA levels fluctuated between the wild-type and mutant groups.

### Ubiquitination of ORF7a lessens its interaction with BclXL and suppresses its aggregation on the ER.

**FIG 6:**
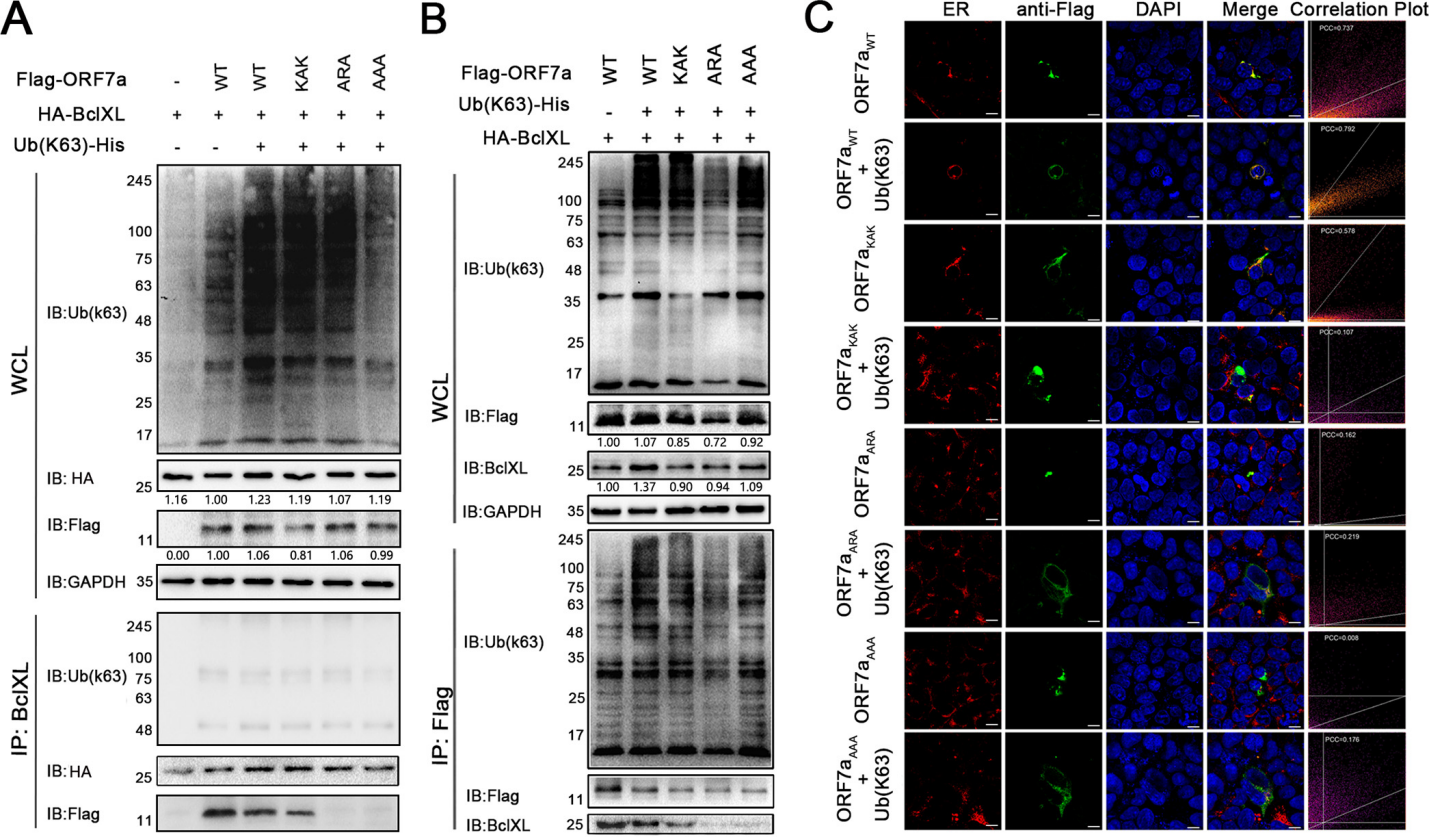
Ubiquitination of ORF7a lessens its interaction with BclXL and suppresses its aggregation on the ER. (A) HEK293 cells were cotransfected with pCAG-Flag-ORF7a (or the mutants), pCAG-HA-BclXL, and pCAG-His-Ub-K63 plasmids for 24 h. The cells were lysed, and the lysates were immunoprecipitated with an anti-BclXL antibody. Immunoprecipitates and WCLs were analyzed via WB with the indicated antibodies. The gray value was calculated and normalized by the GAPDH using ImageJ software. (B) HEK293T cells were cotransfected with Flag-tagged ORF7a (or its mutants) and pCAG-His-Ub-K63 plasmids for 24 h, and the cell lysates were collected for binding with BclXL *in vitro*. After ubiquitination, the interaction between Flag-ORF7a and HA-BclXL was detected by IP using an anti-Flag antibody. The gray value was calculated and normalized by the internal control. (C) The localization of Flag-ORF7a and its mutants was assayed using an IFA and observed by confocal microscopy. Wild-type ORF7a and the mutants were coexpressed with the ubiquitin chain in the cells for 24 h.

It has been reported that ubiquitination of ORF7a at Lys119 promotes antagonism of the interferon response (25). We wondered whether the ubiquitination of ORF7a affects its interaction with BclXL and cell fate. The K63 ubiquitin (Ub [K63]), wild-type ORF7a or its mutants, and BclXL were coexpressed in HEK 293T cells, and the interaction was accompanied by ubiquitination detected by immunoprecipitation. The results in Fig. 6A show that the binding of wild-type and mutated ORF7a to BclXL has reduced in the presence of Ub (K63) coexpression compared to the absence of the ubiquitin chain. Then, Ub (K63), wild-type ORF7a, or mutants were coexpressed in HEK 293T cells, and the interaction of remaining ORF7a after polyubiquitination with BclXL *in vitro* was detected. The results (Fig. 6B) show that ubiquitination of ORF7a_ARA_ was reduced relative to that of the wild-type protein and the other two mutants in the presence of Ub (K63) (WCL in Fig. 6B), and the monomers of wild-type ORF7a or its variations adsorbed by magnetic beads were reduced (IP in Fig. 6B). BclXL decreased in the immunoprecipitation assay (IP in Fig. 6B). In addition, it is worth noting that coexpression of ubiquitin in mutated ORF7a-positive cells changed the subcellular localization of ORF7a on the ER (Fig. 6C), especially in the ORF7a_ARA_ and ORF7a_AAA_ mutants.

### Ubiquitination of ORF7a interrupts ER stress and inhibits apoptosis.

**FIG 7:**
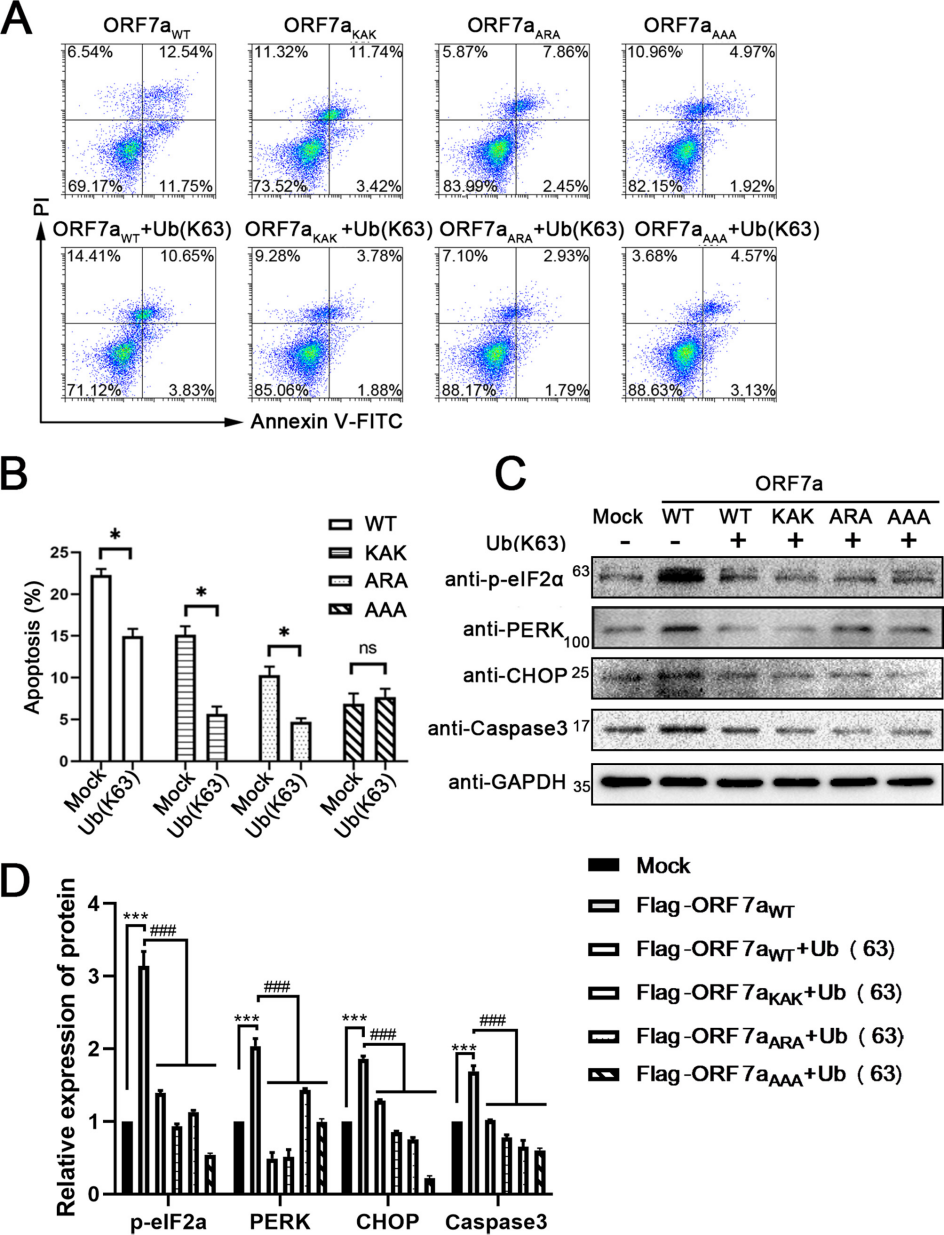
Ubiquitination of ORF7a interrupts ER stress and inhibits cell apoptosis. (A) Apoptosis was measured using flow cytometry at 24 h after transfection with pCAG-Flag, pCAG-Flag-ORF7a, or its mutants (pCAG-Flag-ORF7a_KAK_, pCAG-Flag-ORF7a_ARA_, or pCAG-Flag-ORF7a_AAA_) combined with pCAG-His-Ub-K63 plasmid. (B) The percentage of cell apoptosis was quantified from three independent measurements. (C) PERK, p-eIF2α, CHOP, cleaved Caspase3, and GAPDH in Vero E6 cells were detected by Western blotting at 24 h after coexpression with ubiquitin chain and Flag-ORF7a or its mutants. (D) Gray values of PERK, p-eIF2α, CHOP, and Caspase3 were calculated for three independent experiments using ImageJ. *, *P* < 0.05; ***, *P* < 0.001, compared with the mock group. ##*#*, *P* < 0.001, compared with the WT and no Ub group (one-way ANOVA and Tukey's *post hoc* test). ns. indicates no significant difference.

**FIG 8:**
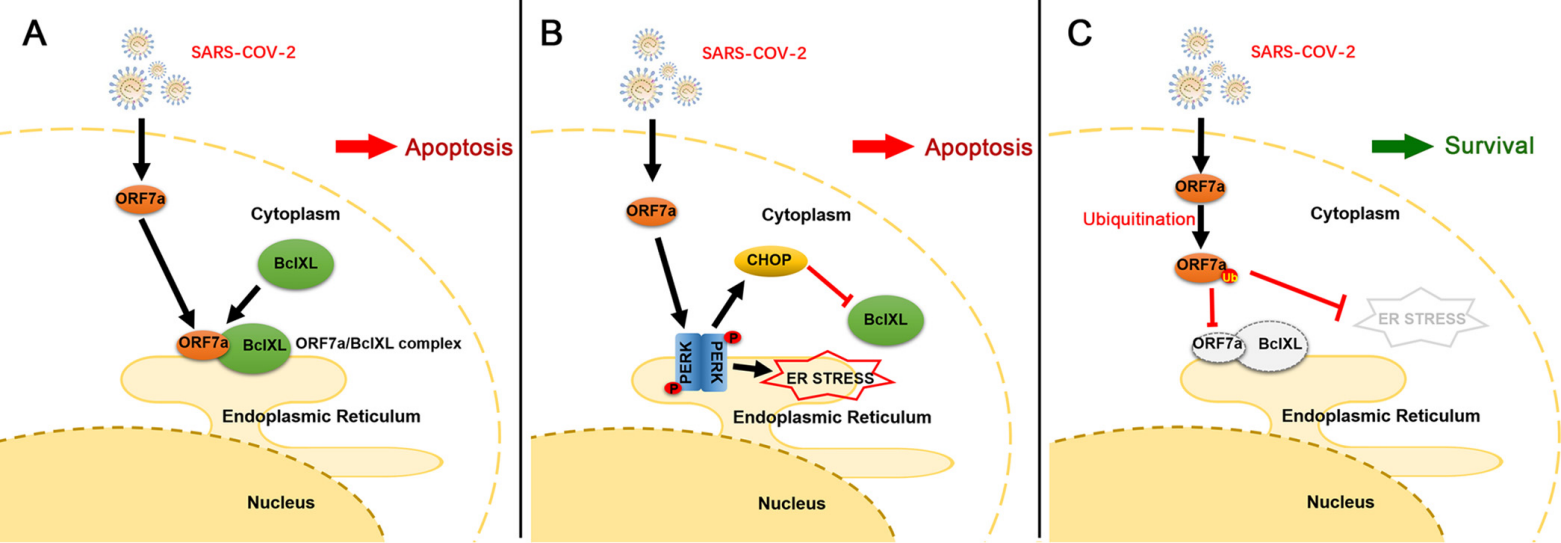
ORF7a interacts with BclXL and activates ER stress, and ubiquitination of ORF7a prevents the above processes. (A) ORF7a recruits BclXL to the ER membrane through the C-terminal amino acids and weakens BclXL to inhibit cell apoptosis. (B) ORF7a induces PERK dimerization and activates ER stress in cells. CHOP inhibits endogenous BclXL, but ER stress leads to apoptosis. (C) The ubiquitination of ORF7a reduces the interaction with BclXL on ER and attenuates the activation of ER stress, rescuing cells.

Coexpression of Ub (K63) and the wild-type ORF7a or its mutants also significantly reduced the apoptotic rate of HEK 293T cells, except the ORF7a_AAA_ mutant (Fig. 7A and B), indicating that ubiquitination of ORF7a at Lys117 and Lys119 can compete with the binding of BclXL, which plays a role in inhibiting cellular apoptosis. We also monitored the ER stress response of cells after the expression of ORF7a and the ubiquitin chain. The results showed that ORF7a upregulated the ER stress transducer PERK, and its effectors p-elF2α, CHOP, and Caspase 3 were also increased (Fig. 7C and D). The addition of the ubiquitin chain reduced all the above markers in the wild-type ORF7a and mutant groups (Fig. 7C and D; Fig. S1B and D). Combined, the results shown in Fig. 6 and 7 indicate that the ubiquitination of ORF7a leads to a reduction in the interaction of ORF7a with BclXL and localization to the ER, resulting in attenuated activation of ER stress and finally rescuing the cells (Fig. 8).

## DISCUSSION

SARS-CoV-2-encoded accessory proteins play important roles in regulating viral replication and antagonizing the host immune system, which determines the fate of host cells. Ren et al. (26)reported that SARS-CoV-2 ORF3a possesses the ability to cause apoptosis in cells by activating the extrinsic apoptotic pathway. ORF3a also inhibits autophagic lysosome formation by blocking SNARE complex assembly, leading to lysosomal damage (27). SARS-CoV ORF8 has important functions, including apoptosis and antagonizing the interferon (IFN) signaling pathway (28, 29). Moreover, our group reported that SARS-CoV-2 ORF7b could mediate TNF-α-induced apoptosis (21).

BclXL, a member of the Bcl-2-related protein family, plays an important biological role in regulating apoptosis and maintaining homeostasis in healthy cells and during viral infection. Yang et al (17) reported that the SARS-CoV envelope protein induces apoptosis in Jurkat T cells and could be inhibited by overexpression of BclXL. In addition, ORF7a from SARS-CoV triggers cell apoptosis by directly interfering with the antiapoptotic protein BclXL (7). This study demonstrated that SARS-CoV-2 ORF7a recruits BclXL through the C-terminal amino acids Lys117 and Lys119 (Fig. 2) and aggregates on the ER membrane (Fig. 4), resulting in the activation of ER stress. To remove large amounts of foreign protein accumulated in the ER and as part of the mechanism of cell stress, host cells tend to undergo death.

We know that BclXL plays a role in the mitochondria, while Tan et al. (7) found that endogenous BclXL protein mainly localizes at other organelles when SARS-CoV ORF7a was overexpressed, indicating that ORF7a from both SARS-CoV and SARS-CoV-2 can change the subcellular location of BclXL. We questioned how the physiological activity of ORF7a captures BclXL to the ER. At the early stage of ER homeostasis disturbance, unfolded or misfolded proteins accumulate in the ER lumen, resulting in ER stress. Then, cells activate controlled regulatory programs, known as the unfolded protein response (UPR), to rescue the normal function of the ER (30). Ultimately, apoptosis will occur to eliminate the stressed cells if ER stress is sustained, and cells fail to reestablish the proper ER protein folding capacity (31). Several pathologies, including metabolic disease, cancer, cardiovascular disease, inflammation, and viral infection, are associated with ER stress, promoting cell death (32–35). ORF7a accumulates in the ER lumen (Fig. 4), functions as a misfolded protein, and disrupts the endoplasmic reticulum's homeostasis, leading to the ER stress response. Prolonged activation of ER stress initiates cell apoptosis via upregulation of CHOP (35). ORF7a expression induced CHOP upregulation (Fig. 5), which inhibited the expression of the antiapoptotic protein BclXL (Fig. 3). CHOP also increases the expression of BAK and BAX, which causes the release of cytochrome c (Cyt-C) through a mitochondria-dependent pathway (35).

Ubiquitination can modulate protein stability, specificity, and affinity to adjust the positions of proteins in an interaction network (36). In virus-host interactions, viruses and hosts utilize a rich source of universal ubiquitination programs to compete, antagonize, or adjust the immune system (36). Numerous well-characterized regulations mediated by ubiquitination have been implicated in the virus replication cycle or the progression of the innate immune response, such as the IFN signaling pathway. HTLV-encoded tax and HBV-encoded polymerase reduce the K63 polyubiquitin chain and block IFN production (37, 38). TOSV expresses an E3 ubiquitin ligase that adds a K48-linked ubiquitin chain to RIG-I to target it for proteasomal degradation (39). SARS-CoV codes a papain-like viral protease nsp3 to mimic deubiquitinases and remove polyubiquitin chains from RIG-I (40). Recently, Cao et al. (25) reported that ubiquitination of SARS-CoV-2 ORF7a usurps the host ubiquitin system to promote antagonism of the type I IFN response. Here, we found that the intracellular ubiquitination of ORF7a and interaction with BclXL occur simultaneously (Fig. 6A). The increased ubiquitination of ORF7a in the WT+Ub (K63) sample of WCL and decreased flag-ORF7a in the elution mix compared to no Ub (K63) WT sample indicated that ORF7a is more ubiquitinated and has a stronger effect on fighting against the interaction with BclXL and diminishing the ER stress. We also demonstrated that ubiquitination of ORF7a reduced the ER stress response and the apoptosis rate (Fig. 7), illustrating that ORF7a utilizes the ubiquitination system to allow host cells to survive longer and facilitate virus reproduction.

The evidence highlights the important roles of E2 and E3 enzymes in mediating spatial and temporal control of ubiquitination (41). We believe that the ubiquitination of ORF7a is regulated by the ubiquitin-proteasome system, but its interaction with BclXL does not need other cellular factors. The membrane-spanning really interesting new gene (RING) finger protein 183 (RNF183) is a classic E3 ligase that localizes to the ER, interacts with BclXL, and polyubiquitinates BclXL for degradation to prolong ER stress (42). In our study, ORF7a was found to have a similar function, which interacts with BclXL and induces the downregulation of endogenous BclXL through CHOP. The difference is that ORF7a activates cellular ER stress, and the host eliminates infected cells through apoptosis to prevent virus spread. Cells adopt a special ubiquitination code to protect against viral infection (43), while the ubiquitination of ORF7a eliminates the effect of host cells on apoptosis, indicating that ORF7a manipulates and exploits the ubiquitin system to redirect cellular pathways in favor of virus survival. Further experimental verification is needed to answer how the interaction or ubiquitination of ORF7a is determined.

We first identified that SARS-CoV-2 ORF7a interacts with BclXL in the ER and actives ER stress. The host initiates programmed death to remove virus-infected cells. However, ORF7a utilizes the ubiquitin system to counteract cell apoptosis, rescuing the cell fate and facilitating virus reproduction (Fig. 8). Due to the COVID-19 epidemic prevention and biosecurity, a limitation of our study is that the SARS-CoV-2 infection was greatly limited. We next plan to perform *in vitro* and *in vivo* infection experiments to establish connections as conditions permit. Our findings reveal that the accessory protein ORF7a may be a potential target for strategies to minimize the COVID-19 pandemic.

## MATERIALS AND METHODS

### Cells and transfection.

HEK 293T and Vero E6 cells were cultured at 37°C in a 5% CO_2_ atmosphere with Dulbecco's modified Eagle medium containing 10% fetal bovine serum (Gibco). Cells were transfected with different plasmids before being seeded into 12-well plates or 100-mm dishes for incubation overnight. The stable HEK 293T-ORF7a cell line was constructed by our team and stored in liquid nitrogen. Lipofectamine 2000 (Thermo Scientific) was used for transient transfection of HEK 293T cells according to the manufacturer's instructions.

### Construction of mutant plasmids.

The wild-type *Orf7a* fragment was synthesized by GENEQIZ (China) and cloned into a pCAGGS-Flag vector through the restriction endonucleases XhoI and *Sac*II. The sequences with the point mutations KAK, ARA, and AAA were amplified using KOD plus polymerase (Toyobo), and the wild-type *Orf7a* fragment was used as the template. The primer pairs are listed as follows: KAK mutant forward, 5′-CACTCAAAGCAAAGACAGAATAGCTCGAGATCA-3′; KAK mutant reverse, 5′-TGATCTCGAGCTATTCTGTCTTTGCTTTGAGTG-3′; ARA mutant forward, 5′-ACTCGCAAGAGCGACAGAATAGCTCGAGATAA-3′; ARA mutant reverse, 5′-TTATCTCGAGCTATTCTGTCGCTCTTGCGAGT-3′; AAA mutant forward, 5′-ACTCGCAGCAGCGACAGAATAGCTCGAGATAA-3′; AAA mutant reverse, 5′-TTATCTCGAGCTATTCTGTCGCTGCTGCGAGT-3′; Lenti-ORF7a forward, 5′-CGGCGGATCCATGAAAATTATTCTTTTC-3′; lenti-ORF7a reverse, 5′-TCGGACGCGTAATTCTGTCTTTCTTTTGAG-3′. All recombinant constructs were screened by PCR and confirmed by DNA sequencing.

### Western blotting.

After treatment, whole cells were lysed with radioimmunoprecipitation assay (RIPA) buffer (Solarbio, R0010) at 4°C for 10 min, and the total proteins were quantified using a bicinchoninic acid (BCA) protein assay kit (Beyotime, P0010S). The proteins were separated via SDS-PAGE and transferred to polyvinylidene fluoride (PVDF, Millipore) membranes. After blocking with 5% skimmed milk, the membranes were incubated with primary antibodies overnight at 4°C. The primary antibodies included monoclonal anti-FLAG M2 (Sigma–Aldrich, F1804, 1:5,000), monoclonal anti-HA tag (Abcam, ab13834, 1:5000), anti-BAX (Abcam, ab53154, 1:5,000), anti-BclXL (ProteinTech, 66020-1-Ig, 1:2,000), anti-Caspase 9 (ProteinTech, 10380-1-AP, 1:1,000), anti-PERK (Abcam, ab65142, 1:500), anti-phosphate eIF2α (Abcam, ab214434, 1:1,000), anti-CHOP (Abcam, ab11419, 1:2,000), anti-Caspase 3 (Abcam, ab208161, 1:1,000), anti-PARP1 (Invitrogen, 436400, 1:2,000) and rabbit polyclonal anti-GAPDH (ProteinTech, 60004-1-Ig, 1:2,000). Following three washes, the membranes were probed with horseradish peroxidase-coupled secondary antibodies. Finally, the membranes were exposed, and signals were recorded using Image Lab software (Bio-Rad) after incubation with enhanced chemiluminescence (ECL) reagents (Beyotime, P0018FM). The relative band intensities were quantified using ImageJ software (V 1.8).

### Reverse transcription and quantitative real-time PCR.

Total RNA was extracted using TRIzol reagent (Thermo Scientific, 15596026) after the cells were washed three times with phosphate-buffered saline (PBS). Complementary DNA (cDNA) was synthesized using a PrimeScript RT reagent kit with gDNA eraser (TaKaRa, RR047B) and an oligonucleotide (dT) primer. Quantitative real-time PCR was performed using BlasTaq 2X qPCR MasterMix (ABM, G892) and the following primer pairs: *DDIT3* forward, 5′-ATCCAACTGCAGAGATGGCA-3′ and reverse, 5′-CAGGGTCAAGAGTGGTGAAGA-3′; *QRICH1* forward, 5′-CTGCAAGCAGCTCAGATCCAG-3′ and reverse, 5′-TCTGGATTTGGATCTGCTGA-3′; *GAPDH* forward, 5′-TGGAAGGACTCATGACCACA-3′ and reverse, 5′-AGGCAGGGATGATGTTCTGG-3′. The thermocycling conditions were set as follows: 95°C for 30 s, followed by 40 cycles at 95°C for 5 s and 60°C for 30 s. Each reaction was set up in triplicate, and the data were collected from three independent experiments. The mRNA levels of target genes were normalized to GAPDH levels using the 2^−ΔΔCt^ method (44).

### Apoptosis assay.

Cellular apoptosis was detected using an annexin V-FITC apoptosis staining/detection kit (Abcam, ab14085). HEK 293T cells transfected with plasmids encoding wild-type Flag-ORF7a or its mutants for 24 h in 12-well plates were collected and washed thrice with PBS, and 500 μL of binding buffer was added to resuspend the cells. Then, the cells were mixed with 5 μL of annexin V-FITC and 2 μL of propidium iodide. After incubation in the dark for 10 min at room temperature, the percentages of apoptotic cells were detected by flow cytometry using a CytoFLEX system (Beckman Coulter). Each treatment was repeated independently three times.

### Coimmunoprecipitation.

Whole cells from 100-mm dishes were lysed using RIPA buffer (Solarbio, R0010) containing 50 mM Tris (pH 7.4), 150 mM NaCl, 1% NP-40, 0.25% sodium deoxycholate, and 1 mM protease inhibitor PMSF (Thermo Scientific, 36978) for 10 min at 4°C. Immunoprecipitation was performed using an anti-Flag M2 (Sigma, F1084) or anti-BclXL (ProteinTech, 66020-1-Ig) antibody with Pierce protein A/G magnetic beads (Thermo Scientific, 88802) at 4°C for 3 h. The precipitated proteins were washed three times with washing buffer and then eluted from the beads with elution buffer (50 mM sodium phosphate, pH 8.0, 300 mM sodium chloride, 300 mM imidazole). The immunocomplexes were detected by Western blotting using the related antibodies.

### Immunofluorescence and confocal microscopy.

HEK 293T cells transfected with plasmids or stable HEK 293T-ORF7a cell lines were immobilized using 4% paraformaldehyde and permeabilized with Triton X-100 for 10 min. After washing with PBS and blocking with 5% skimmed milk, the cells were incubated with primary antibodies targeting Flag/HA-fused proteins or organelles overnight at 4°C. After three washes with PBS, the cells were incubated with FITC-conjugated IgG or Alexa Fluor 647-conjugated IgG for 1 h at room temperature and then stained with DAPI for 20 min. The fluorescence signals were recorded via confocal microscopy (Nikon, PCM-2000).

### *In vivo* ubiquitination and binding assay.

HEK 293T cells were cotransfected with plasmids expressing wild-type or mutant Flag-ORF7a, HA-BclXL, and 6×His-tagged ubiquitin for 24 h. The cells were collected, washed with PBS, and lysed in RIPA buffer supplemented with a protease inhibitor in two pellet volumes. The lysates were sonicated and centrifuged at 4°C for 15 min. The supernatants were incubated with protein A/G magnetic beads and 5 μL anti-BclXL antibody at 4°C for 3 h. After extensive washing, the bound lysates were eluted with elution buffer and separated via SDS-PAGE. Then, ubiquitination and ORF7a-BclXL binding were detected via Western blotting using anti-Ub(K63) or anti-Flag antibodies.

### *In vitro* binding assay.

HEK 293T cells in 100-mm wells were cotransfected with plasmids expressing wild-type or mutant Flag-ORF7a and a 6×His-tagged ubiquitin chain for 24 h, and cells in another well were transfected to express HA-BclXL. Then, the cells were washed and lysed with RIPA buffer, and the lysates were sonicated and centrifuged at 4°C and 16,000 *g* for 15 min. The supernatants were incubated with equivalent lysates containing HA-BclXL at 4°C overnight. Then, an anti-Flag antibody was used to pull down the BclXL-ORF7a mix *in vitro*, and the interaction of ORF7a with BclXL after ubiquitination was detected via Western blotting using an anti-BclXL antibody.

### Statistical analyses.

The data are reported as the mean ± standard deviation of three independent experiments. Statistical analysis was performed using GraphPad Prism 5 and SPSS (v19.0). The difference between the two groups was tested using an unpaired Student's *t* test, and multiple groups were compared via one-way ANOVA and Tukey's *post hoc* test. */# indicates a *P* value less than 0.05; **/## indicates a *P* value less than 0.01; ***/### indicates a *P* value less than 0.001; and ns. indicates no significant difference.
