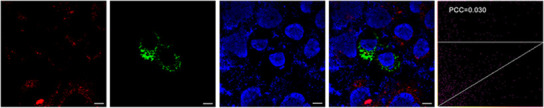# Erratum for Liu et al., “Ubiquitination of SARS-CoV-2 ORF7a Prevents Cell Death Induced by Recruiting BclXL To Activate ER Stress”

**DOI:** 10.1128/spectrum.03670-23

**Published:** 2023-12-07

**Authors:** Zhixin Liu, Yanan Fu, Yanping Huang, Feng Zeng, Jingjing Rao, Xiao Xiao, Xiaoguang Sun, Hao Jin, Jian Li, Jing Yang, Weixing Du, Long Liu

Volume 10, no. 6, e01509-22, 2022, https://doi.org/10.1128/spectrum.01509-22. Page 8, Fig. 6C, bottom row: Incorrect images were inserted by mistake. The ORF7a_AAA_+Ub(K63) row should appear as shown below. There are no corresponding changes to the text or figure legend. This change does not alter the results or conclusions of the study.

**Fig 6:**