# Supplementary material for: Ubiquitination of SARS-CoV-2 ORF7a Prevents Cell Death Induced by Recruiting BclXL To Activate ER Stress
Source: Microbiol Spectr. 2022 Nov 3;10(6):e01509-22. doi: 10.1128/spectrum.01509-22 (PMC9769937; doi:10.1128/spectrum.01509-22)
Supplement: Supplemental file 1 — Fig. S1. Download spectrum.01509-22-s0001.pdf, PDF file, 0.1 MB [file spectrum.01509-22-s0001.pdf]

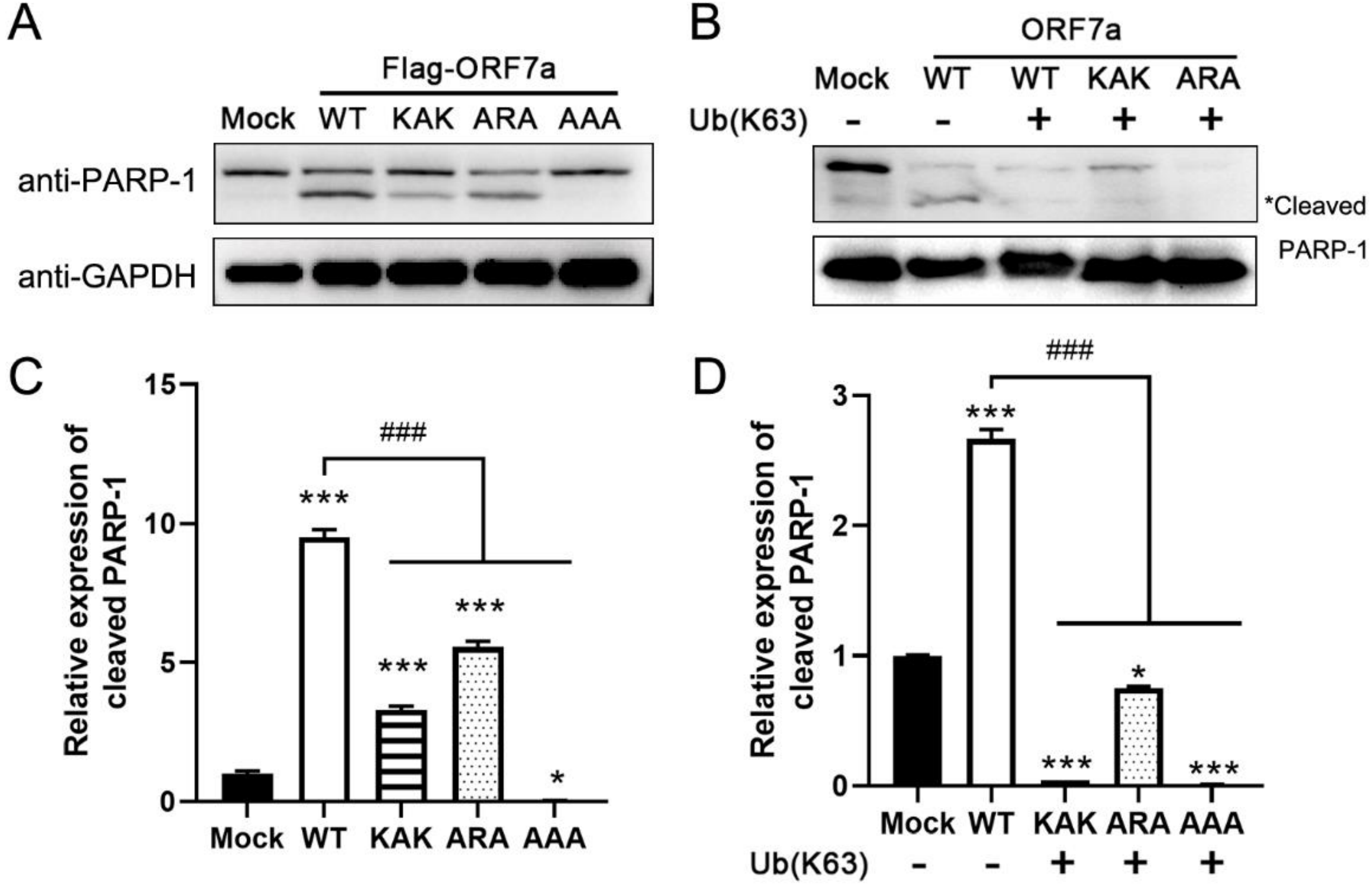

Fig. S1 The cleaved PARP-1 in cells was detected after treatment with wild-type / mutant ORF7a and ubiquitin chain. (A) Cleaved PARP-1 and GAPDH in Vero E6 cells was detected by Western blotting at 24 h after transfection with pCAG-Flag, pCAG-Flag-ORF7a, or mutants (pCAG-Flag-ORF7a$_{KAK}$, pCAG-Flag-ORF7a$_{ARA,}$ or pCAG-Flag-ORF7a$_{AAA}$). (B) Cleaved PARP-1 and GAPDH in Vero E6 cells were detected by Western blotting at 24 h after co-expression with ubiquitin chain and Flag-ORF7a or its mutants (Flag-ORF7a$_{KAK}$ or Flag-ORF7a$_{ARA}$). (C&D) Western blotting results were calculated for three independent experiments from A & B using ImageJ. * $P < 0.05$, *** $P < 0.001$, compared with the Mock group; ### P < 0.001, compared with the WT or & no Ub group (one-way ANOVA and Tukey's post-hoc test).
